# Predicting Duodenal Cancer Risk in Patients with Familial Adenomatous Polyposis Using Machine Learning Model

**DOI:** 10.5152/tjg.2023.22346

**Published:** 2023-10-01

**Authors:** Sami Akbulut, Zeynep Küçükakçalı, Cemil Çolak

**Affiliations:** 1 Department of Surgery, İnönü University Faculty of Medicine, Malatya, Turkey; 2 Department of Public Health, İnönü University Faculty of Medicine, Malatya, Turkey; 3 Department of Biostatistics and Medical Informatics, İnönü University Faculty of Medicine, Malatya, Turkey

**Keywords:** Familial adenomatous polyposis, duodenal cancer, gene mutations, genomics, machine learning, XGboost

## Abstract

**Background/Aims:**

The aim of this study was to both classify data of familial adenomatous polyposis patients with and without duodenal cancer and to identify important genes that may be related to duodenal cancer by XGboost model.

**Materials and Methods:**

The current study was performed using expression profile data from a series of duodenal samples from familial adenomatous polyposis patients to explore variations in the familial adenomatous polyposis duodenal adenoma–carcinoma sequence. The expression profiles obtained from cancerous, adenomatous, and normal tissues of 12 familial adenomatous polyposis patients with duodenal cancer and the tissues of 12 familial adenomatous polyposis patients without duodenal cancer were compared. The ElasticNet approach was utilized for the feature selection. Using 5-fold cross-validation, one of the machine learning approaches, XGboost, was utilized to classify duodenal cancer. Accuracy, balanced accuracy, sensitivity, specificity, positive predictive value, negative predictive value, and F1 score performance metrics were assessed for model performance.

**Results:**

According to the variable importance obtained from the modeling, ADH1C, DEFA5, CPS1, SPP1, DMBT1, VCAN-AS1, APOB genes (cancer vs. adenoma); LOC399753, APOA4, MIR548X, and ADH1C genes (adenoma vs. adenoma); SNORD123, CEACAM6, SNORD78, ANXA10, SPINK1, and CPS1 (normal vs. adenoma) genes can be used as predictive biomarkers.

**Conclusions:**

The proposed model used in this study shows that the aforementioned genes can forecast the risk of duodenal cancer in patients with familial adenomatous polyposis. More comprehensive analyses should be performed in the future to assess the reliability of the genes determined.

**Main Points**

- The proposed model can classify the data obtained from familial adenomatous polyposis patients with and without duodenal cancer and identify possible important genes associated with duodenal cancer.
- Accuracy, balanced accuracy, sensitivity, specificity, positive predictive value, negative predictive value, and F1 score metrics for the XGboost were extremely high in each of the cancer-adenoma, adenoma–adenoma, and normal-adenoma comparisons. It was observed that the discriminating power of the proposed model was quite successful.
- Genes determined with variable importance values based on the proposed model for each comparison can be used as potential biomarkers.

## Introduction

Familial adenomatous polyposis (FAP) is a precancerous autosomal dominant condition induced by a mutation in the adenomatous polyposis coli (APC) gene with a population prevalence of 1:10 000.^1^ The FAP is recognized with many adenomatous of the gastrointestinal mucosa and a definite set of extraintestinal lesions encompassing several organs and tissues.^2^ The FAP is characterized by germline pathogenic variants in the APC gene known as one of the tumor suppressor genes located on the long arm, in the 5q21-q22 region of chromosome 5.^3^ The APC is involved in cell cycle modulation via regulating the beta-catenin location and cellular polarity. The APC also participates in the maintenance of T-cell populations in the lamina propria, which have an impact on chronic inflammation and tumor growth.^4,5^

The formation of hundreds to thousands of adenoma polyps in the rectum and colon is the most visible sign of FAP. The FAP often occurs in puberty and with nearly unavoidable progress to colorectal cancer (CRC) in the fourth decade of life. Approximately 70% to 80% of all tumors are found on the left side of the colon. The FAP is best known for the adenomatous polyps that bear its name; however, patients are more likely than the general population to develop other intestinal and extraintestinal manifestations, including fibromas, fibromatosis, gastric fundic gland polyps, duodenal polyps, nasal angiofibromas, thyroid carcinomas, congenital hypertrophy of the retinal pigment epithelium, hepatoblastomas, brain tumors, and pancreatobiliary tumor.^2^ The major hallmark of FAP is colorectal adenomatous polyposis, which spreads all through the colorectum, beginning in childhood and teenage years. By the age of 15, roughly 50% of FAP patients have a colorectal adenoma, and this rate climbs to 95% by the age of 35. The lifetime risk of colorectal carcinoma is almost 100%. If these adenomatous polyps are not treated, it is virtually inevitable that they will turn into invasive carcinoma in patients aged 35-40 years on average.^2^ The duodenum is the second most prevalent location of FAP-associated adenomatous polyps, and it occurs in 30% to 70% of FAP patients. Duodenal/periampullary carcinoma is the second largest cause of mortality in FAP patients, after CRC, with a lifetime risk of development similar to CRC of nearly 100%. Duodenal adenomas of FAP usually occur in the second and third (vertical and horizontal) portions of the duodenum.^6,7^

Genomic technology is a science that uses information technology to process and store its outputs. It was established as a result of breakthroughs in automation and bioinformatics. Research in practically every department of medicine (oncology, pharmacology, immunology, biochemistry, microbiology, and so on) can be carried out with the proper configuration of genetic technology.^8^ It allows for research into cancerization and prognosis prediction, medication response prediction and tailored drug creation, immune response nature, and even transplantation outcome prediction through comparative studies. Next-generation sequencing (NGS) has enabled recent advancements in the analysis of genomic alterations in cancer research and therapeutic practice.^9,10^ Simultaneous analysis of multiple differentially expressed genes (DEGs) is essential for life science researchers’ success in the fields of molecular completeness, functional genomics, drug target discovery, and pharmacogenomics. Comparing the expression levels of the investigated genes between normal and diseased tissue provides important clues for understanding the pathogenesis of the disease. Ultimately, identifying changes in disease-related gene expression levels will enable the identification of new treatments and diagnoses in the future.^11^

Epigenetics has emerged as a promising field for diagnosing and treating common illnesses (e.g., FAP, cancer, etc.), with various epimarkers and epidrugs currently licensed and in clinical usage. As a result, it may become a chance to discover new disease mechanisms and treatment targets for rare diseases.^12^

Machine learning (ML) is an artificial intelligence (AI) subfield that employs data-driven learning to create forecasts about fresh data. The researchers’ goal is to enable computers to recognize complicated patterns and make data-driven decisions.^13^ Due to the accessibility of big data and greater computer power, ML algorithms have attained high performance in a wide range of circumstances over the previous decade.^14^ In recent years, ML approaches have become one of the most commonly utilized technologies in disease diagnosis and clinical decision support systems, with several applications. When it comes to disease prediction categorization, ML approaches are commonly applied.^15,16^ Machine learning is the cornerstone of implementations in genetic disorders diagnosis, early diagnosis of malignant diseases, and pattern recognition in diagnostic imaging, all of which have a wide range of health-related applications.^17^ Extreme Gradient Boosting (XGBoost) is an ML approach that relies on the gradient augmented approach and decision trees that have grown in popularity due to their outstanding classification performance in both data science and remote sensing sectors.^18,19^ The fundamental reason for this method’s success is the objective function it employs in the learning process. It is made up of the goal function, the loss function, and the regularization terms. The loss function computes the difference between the model’s predicted and actual class values.^20,21^

This study aimed to both classify data of FAP patients with and without duodenal cancer and to identify important genes that may be related to duodenal cancer by XGboost model.

## MATERIALS and Methods

### Dataset

The XGboost was applied to open-access transcriptome data of FAP patients with and without duodenal cancer. Open-access dataset was obtained from https://www.ncbi.nlm.nih.gov/geo/query/acc.cgi?acc=GSE111156. The dataset used consists of normal, adenomatous, and cancerous tissues attained from 12 FAP patients with duodenal cancer (FAP cases; n = 36 specimens), and adenoma tissue gained from 12 FAP patients without duodenal cancer (FAP control; n = 12 specimens). The expression profiles obtained from cancerous, adenomatous, and normal tissues of 12 FAP patients with duodenal cancer and the tissues of 12 FAP patients without duodenal cancer were compared (cancer vs. adenoma, adenoma vs. adenoma, normal vs. adenoma).^22^

### Differential Gene Expression Analysis

Differential expression analysis can be applied to normalized read count data by doing statistical analysis to detect quantitative variations in expression profile levels among experimental groups. For example, we employ statistical testing to evaluate whether or not an observed variation in reading counts for a specific gene is statistically significant, whether or not it is bigger than what would be predicted simply by chance.^23^ As a result, derived from the term differential expression, differential expression analysis aims to validate which genes are expressed at separate levels in different conditions. These genes can provide biological information about the processes impacted by the condition(s) of interest. The determination of such changes may be necessary for the determination of biomarkers of diseases and cancers with some genetic background and, accordingly, in their treatment.

### Feature Selection

In any predictive modeling effort, variable selection is critical. Choosing which data to include is one of the most crucial aspects of constructing a statistical model. Before dealing with very big datasets and models with high computing costs, considerable efficiency can be attained by determining the most valuable aspects of a dataset. The process of detecting features in a data collection that influence the dependent variable is known as feature selection. The process of identifying features in a data collection that impacts the dependent variable is known as feature selection. In addition, models with many characteristics are harder to understand. Important features should ideally be selected before statistical modeling.^24^ Most ML and data mining techniques may be useless when confronted with high-dimensional data. Consequently, these methods generate more effective outcomes when the dimensionality is lowered.^25^ Gene expression data are quite massive. Modeling analysis requires a long time due to the high amount of gene expression datasets, and these data may lead to computing inefficiencies in the study.

A classification method may overfit the training instances and undergeneralize new samples in gene expression datasets with many genes. Many regularization methods such as least absolute contraction and selection operator (LASSO), ridge, and elastic-net have been propounded for model fitting and variable selection in poorly described multiple regressions. Ridge regularization makes predictors go down, which makes parameter estimation more stable. Numerous regression coefficients approach exactly zero after LASSO regularization. This makes it easier for auto-variable selection, which means that only one predictor is chosen from those that are correlated. Elastic-net regularization employs both ridge penalties and LASSO penalties concurrently to get the most out of both. As a result, it offers shrinkage and auto-variable selection, and the ability to handle more effectively the extreme multicollinearity that is common in genome-wide association study (GWAS) analysis.^26-29^

### XGBoost Model

First introduced in 2001 as an effective ML algorithm, Gradient Boosting (GBoost) is a technique that uses boost methods and is an ensemble form of models that can perform regression and classification, often producing poor prediction results such as decision trees.^30,31^ The basic structure of XGBoost, unlike GBoost, is based on the gradient boosting method in addition to decision tree techniques. The first prototype of the XGBoost was developed by Friedman in 2002.^32^ The method has gained a lot of attention in the ML industry after 2 University of Washington researchers, Tianqi Chen and Carlos Guestrin, presented the method at a conference in 2016.

The XGBoost is a well-known algorithm that is employed in the domains of health, energy, finance, and so on. When compared to other algorithms, it has a significant speed and performance advantage. It provides a huge speed and performance advantage over other algorithms. The XGBoost is also 10 times quicker than previous algorithms, with many regularizations that enhance overall performance while reducing overfitting and over-learning. GBoost is an ensemble technique for merging multiple weak classifiers with boosting to create a strong classifier. The strong learner is educated recursively, beginning with a basic learner. GBoost and XGBoost work on the same concepts. The main distinction between them is in the implementation specifics. XGBoost improves performance by regulating the complexity of the trees through the use of various regularization algorithms.

### Modeling

XGBoost was employed in the modeling. Analyses were conducted using the n-fold cross-validation technique. In the n-fold cross-validation approach, the data are separated into n parts before the model is applied to each of the n parts. One of the n components is utilized for testing, while the remaining n-minus-one components are used to train the model. In this work, 5-fold cross-validation was performed for the modeling procedure. As performance assessment criteria, we employed accuracy (ACC), balanced accuracy (B-ACC), sensitivity, specificity, positive predictive value (PPV), negative predictive value (NPV), and F1 score. In addition, variable importance was determined, which offers information on how much the factors assign importance to the outcome variable.

### Statistical Analysis

Data were summarized as mean ± standard deviation based on the variable distribution. Shapiro–Wilk test of normality was employed to determine whether the variables had a normal distribution. Independent-sample *t*-test was employed for statistical analysis. *P* < .05 was deemed statistically significant. IBM Statistical Package for the Social Sciences Statistics 25.0 program (IBM Corp.; Armonk, NY, USA) was employed in the analysis.

## Results

The median age of the entire patients included in the dataset was 48.5 years (min-max = 34-70). There were no statistical differences between FAP cases and FAP control groups in terms of race, age, gender, sulindac/celecoxib use, nor do they differ in terms of dysplasia, size, histology, or polyp number. This information was obtained from the index article.^22^

### Subgroup Analysis Based on Genetic Alterations

#### Cancerous (Familial Adenomatous Polyposis Cases) Versus Adenomatous (Familial Adenomatous Polyposis Control) Tissues:

**Figure 1.**
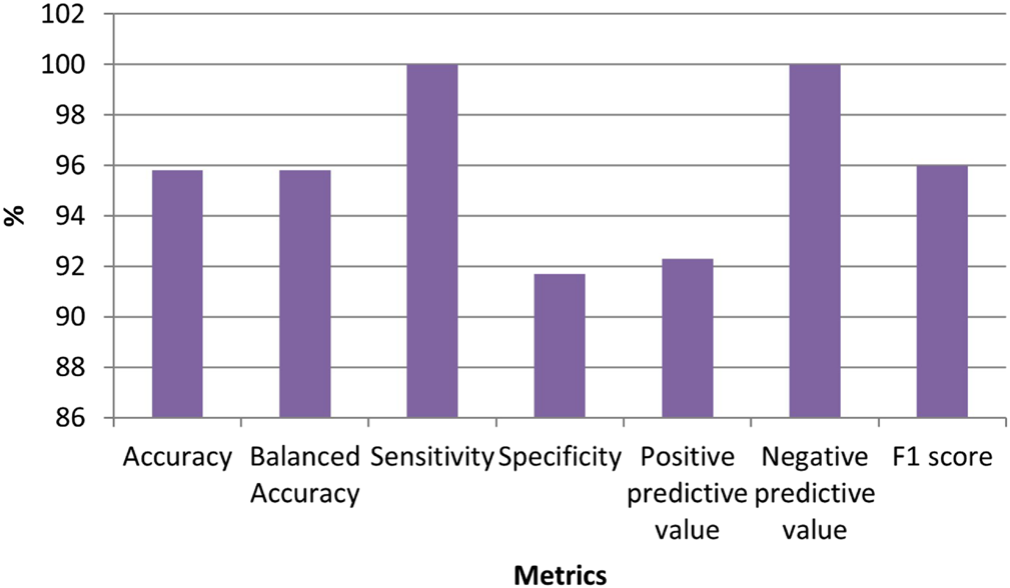
Graph for performance metrics obtained from XGboost models.

**Figure 2.**
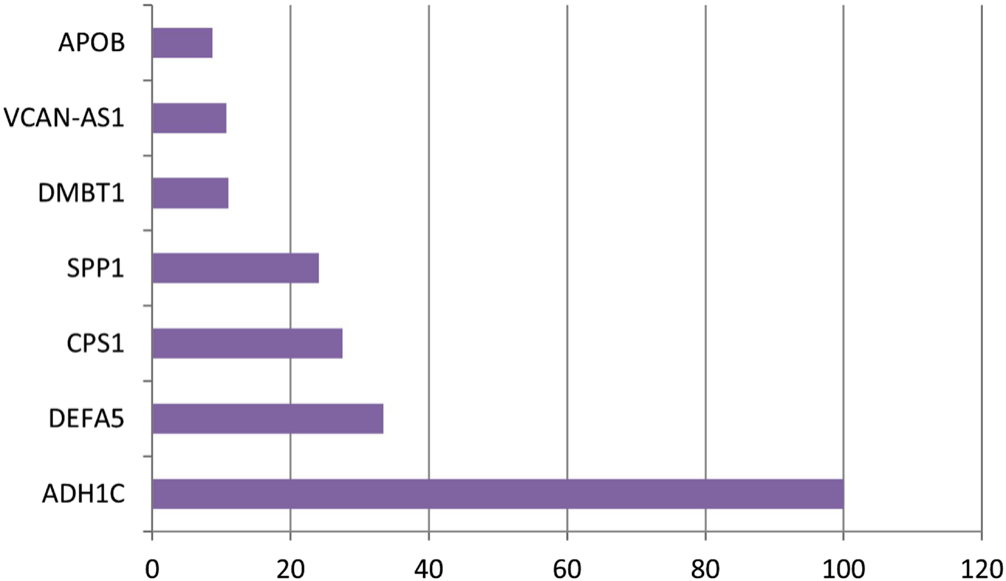
The graph of variable importance values.

Thirteen genes remained in the dataset created by using the Elastic Net technique from the dataset consisting of 70 523 expressed sequence tags (ESTs). Table 1 shows the definition of the dataset established with these selected ESTs as well as the identifiers of the inspected target variable. Table 2 shows the results of the performance metrics derived using the XGboost findings. The ACC, B-ACC, sensitivity, specificity, PPV, NPV, and F1 score calculated from the XGboost were 95.8%, 95.8%, 100%, 91.7%, 92.3%, 100%, and 96%, respectively. The values of performance criteria calculated from the XGboost are plotted in Figure 1. The variable importance in terms of explaining the output variable of the ESTs, which are the input variables, is given in Figure 2.

#### Adenomatous (Familial Adenomatous Polyposis Cases) Versus Adenomatous (Familial Adenomatous Polyposis Control) Tissues:

**Figure 3.**
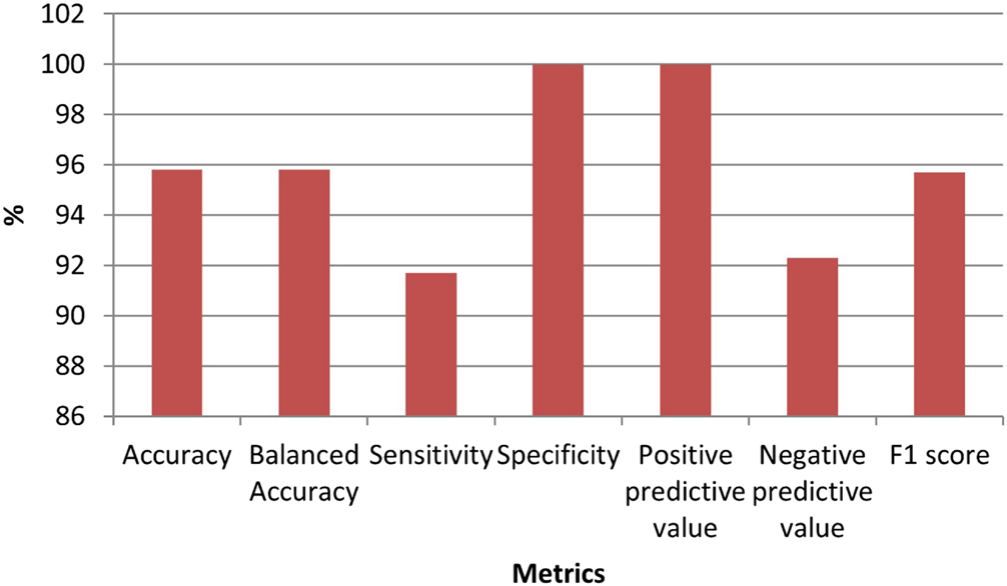
Graph for performance metrics obtained from XGboost models.

**Figure 4.**
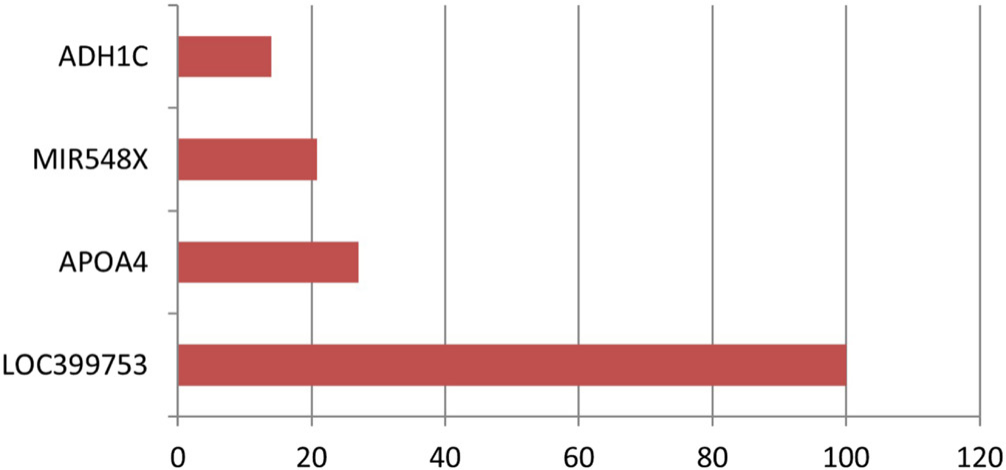
The graph of variable importance values.

Nine genes remained in the dataset created by using the Elastic Net technique from the dataset consisting of 70 523 ESTs. Table 3 shows the definition of the dataset established with these selected ESTs as well as the identifiers of the inspected target variable. Table 4 shows the results of the performance metrics derived using the XGboost findings. The ACC, B-ACC sensitivity, specificity, PPV, N, and F1 score calculated from the XGboost were 95.8%, 95.8%, 91.7%, 100%, 100%, 92.3%, and 95.7%, respectively. The values of performance criteria calculated from the XGboost are plotted in Figure 3. The variable importance in terms of explaining the output variable of the ESTs, which are the input variables, is given in Figure 4.

#### Normal (Familial Adenomatous Polyposis Cases) Versus Adenomatous (Familial Adenomatous Polyposis Control) Tissues:

**Figure 5.**
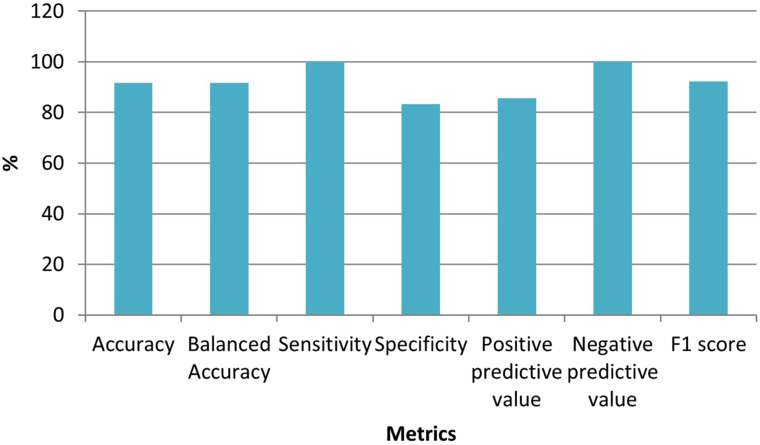
Graph for performance metrics obtained from XGboost models.

**Figure 6.**
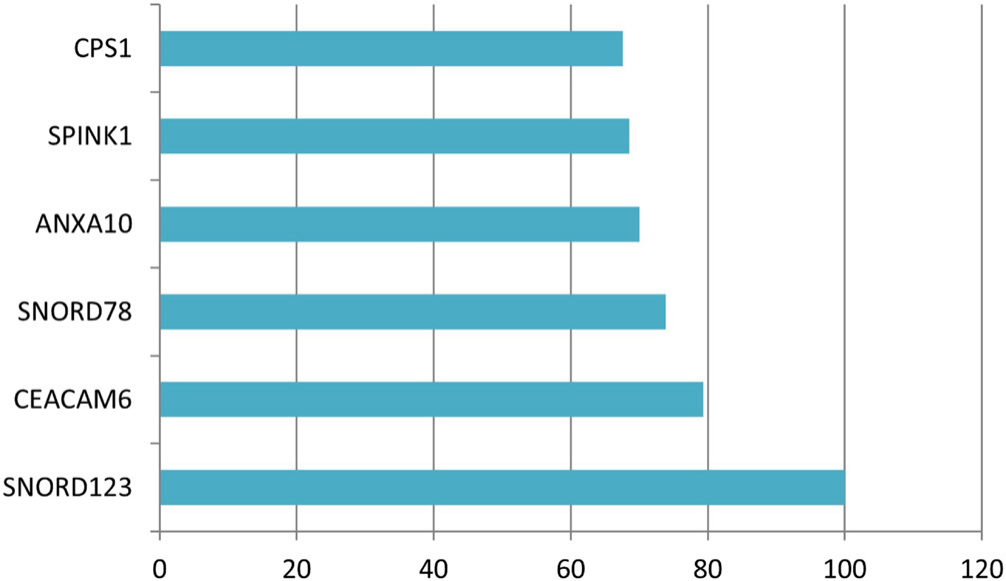
The graph of variable importance values.

Sixteen genes remained in the dataset created by using the Elastic Net technique from the dataset consisting of 70 523 ESTs. Table 5 shows the definition of the dataset established with these selected ESTs as well as the identifiers of the inspected target variable. Table 6 shows the results of the performance metrics derived using the XGboost findings. The ACC, B-ACC sensitivity, specificity, PPV, NPV, and F1 score calculated from the XGboost were 91.7%, 91.7%, 100%, 83.3%, 85.7%, 100%, and 92.3% respectively. The values of performance criteria calculated from the XGboost are plotted in Figure 5. The variable importance in terms of explaining the output variable of the ESTs, which are the input variables, is given in Figure 6.

## Discussion

The FAP is an infrequent autosomal dominant genetic condition caused by many adenomatous polyps that inevitably proceed to colorectal carcinoma if not diagnosed and treated early.^2^ The incidence of FAP is almost 1 in 7000 to 1 in 30 000 births. The FAP is with a high penetrance that impacts both men and women equally and has varying expressivity. The majority of those affected have a family background of FAP symptoms; nevertheless, de novo mutations account for a large portion of cases (about 20%-30%). The severity of both intestinal and extraintestinal disease has been associated with the mutations in certain areas of the APC gene.^2,33,34^

Patients with FAP frequently have numerous colorectal adenomas, and without total prophylactic proctocolectomy, their overall risk of CRC can approach 100%.^35^ Duodenal cancer has risen to become the second-largest cause of mortality among FAP patients. The lifetime risk of developing duodenal cancer in patients with FAP is around 12%, and duodenal adenomas are seen in 65% of patients with FAP.^36,37^ The severity of duodenal adenomas rose with age, according to a major multi-national study that followed 368 FAP patients for a median of 7 years.^38^ The high prevalence and risk of cancer growth, according to the Spigelman staging technique, necessitate continuing monitoring, and screening should begin around the age of 25-30 years. Family identification and following screening methods have greatly lowered morbidity and mortality in duodenal cancer. However, determining the right time for surgery and which endoscopic results imply surgery remains a challenge. The Spigelman scoring method is employed to classify malignant tumors of FAP patients based on the size, morphology, quantity, and dysplasia of duodenal polyps during endoscopy, and mounting evidence shows that this system underestimates the risk of duodenal cancer in FAP patients with duodenal polyposis. As a result, new methods for predicting cancer risk in FAP patients are required. As FAP is a genetic disease, new gene mutations involved in FAP are constantly being identified as research on the disease advances, implying that patients with FAP have a genetic background difference. The etiology of FAP is complicated caused of the accumulation effects of factors such as the patient’s living space, diet, age, and gender, and there are many ambiguities in rehabilitation and treatment options. In light of this genetic background and differences in other factors, the characteristics of FAP should be analyzed, and genomic studies should increase. In addition, revealing biomarkers with therapeutic benefits that may be related to the condition will also be useful in shaping the treatment of the disease, and target-based therapies can be developed.^36^

In the dataset used in this study, gene expression analysis was performed in the samples taken from the duodenal samples of FAP patients diagnosed with duodenal cancer and FAP patients without a history of cancer. In the current study, comparisons of gene expression profiles obtained from tissue samples with cancer, adenoma, and normal from 12 FAP patients with duodenal cancer and adenoma tissue samples from 12 FAP patients without cancer were used. Gene expression data obtained contained 70 523 ESTs.

Gene expression datasets are relatively huge, and modeling with larger data can result in lengthy analytical durations and computational inefficiencies.

For this reason, before modeling with the current data, the most relevant genes that can be connected with the target variable were selected with the Elastic Net method, which can deal more effectively with severe polylinearity, which is common in GWAS analysis. With this method, 13 out of 70 523 ESTs for cancer-adenoma comparison, 9 for adenoma–adenoma comparison, and 16 for normal-adenoma comparison were selected as the genes most associated with the output variable. ACC, B-ACC, sensitivity, specificity, PPV, NPV and F1 score metrics obtained from the XGboost algorithm were found to be high in cancer-adenoma, adenoma-adenoma and normal-adenoma comparisons. According to the variable significance results obtained with XGBoost for cancer-adenoma, adenoma–adenoma, and normal-adenoma comparisons in the current study, ADH1C, DEFA5, CPS1, SPP1, DMBT1, VCAN-AS1, and APOB genes can be used as biomarkers for duodenal cancer patients with FAP for cancer-adenoma comparison. Likewise, considering the variable importance values obtained, LOC399753, APOA4, MIR548X, and ADH1C genes can be used to differentiate duodenal cancer patients with FAP from the adenoma tissues of non-cancer patients with only FAP for adenoma–adenoma comparison. And finally, SNORD123, CEACAM6, SNORD78, ANXA10, SPINK1, and CPS1 genes can be used as biomarkers for normal-adenoma comparison. New methodology (e.g., methods for sequencing single-cell epigenomes) and diagnostics are being developed to integrate epigenetic markers and their tracking in medical practice. Medical epigenetics is already widely used in oncology, with markers for diagnosis, prognosis, and therapy response ratified by the US Food and Drug Administration, as well as epigenetic-based medicines. In neurological, immunological, metabolic, and infectious illnesses, it is also becoming a growing specialty.^12^ From an epigenetic perspective, important and interesting clinical results were determined in the prediction of the related disease in this study. Individualized medicine is closely linked to the collection, processing, and synthesis of information from various “omics” techniques as well as data from patients and healthcare professionals. Machine learning, which is the branch of AI that provides tools “that may be employed to create and train algorithms to learn from and respond to data” can play a substantial role in aiding clinicians in incorporating, evaluating, and handling.^39^ In this context, the clinical findings obtained from this research can shed light on the use of AI in personalized medicine applications.

There have been limited publications on FAP patients who developed duodenal adenocarcinoma. Studies are needed to examine the underlying pathophysiology of the disease in FAP patients who develop duodenal cancer. In one study, the median survival of 16 FAP patients with duodenal cancer was 11 months.^40^ A study classified the same dataset with the support vector machine model, which is one of the ML. According to the results obtained, the classification result correctly classified the FAP patients with and without duodenal cancer.^36^ In another study, genomic and transcriptome profiles of carcinoma in patients with FAP were carried out. Whole-exome, whole-genome, and single-cell RNA sequencing were implemented in the mentioned study on matched adjoining normal tissues, multiregional exemplified adenomas at various levels, and carcinomas from 6 FAP and 1 MUTYH-associated polyposis patients.^41^ In a recent study carried out through whole-exome sequencing, a point variant in the noncoding region in the APC gene was determined.^42^ Another recent study examined the findings of APC gene analyses. The complete coding sequence of the APC gene was analyzed by the Sanger technique to uncover genetic anomalies. Of the 266 cases pooled, pathogenic/possibly pathogenic variants in the APC gene were determined in 73 patients, and variants of unknown importance were identified in 13 patients. Fourteen of these versions were brand new.^43^ In another study, 27 probands with more than 10 colorectal polyps were used. After evaluation of their symptoms and familial backgrounds, the probands were examined for APC and MUTYH mutations using NGS. In the APC gene, 3 novel truncating variations (p.Leu360*, p.Leu1489Phefs*23, and p.Leu912*) were brought to light in 3 unrelated probands.^44^ One study made available 15 novel APC mutations in the Indian FAP cohort and a novel Indian APC mutational hotspot at codon 935.^45^ In the study from which the dataset used in this study was obtained, DEGs in the duodenal adenoma–carcinoma pathway were detected in patients with FAP who developed duodenal cancer and in FAP patients without duodenal cancer.^22^ Identifying such changes may be important for understanding the treatment of duodenal polyposis and detecting cancer markers. With such studies, genes that may have prognostic and therapeutic importance can be identified.

In the study, in which the dataset used in the current study was obtained, DEFA5 and DEFA6 genes were downregulated, and SPP1 genes were upregulated for cancer-adenoma comparison.^22^ In this study, these genes were selected as genes that may be associated with cancer using the feature selection method, and DEFA5 and SPP1 were determined as the most important cancer-related genes according to their variable importance. For the adenoma–adenoma comparison made in the reference article, the CLCA1 and ADH1C genes were downregulated. In this study, these 2 genes were selected in relation to adenoma by the method of feature selection, and at the same time, ADH1C was among the most important genes associated with adenoma according to their variable significance.

This study has a few limitations. First, it is essential to confirm the clinical findings extracted from this study with the results of other clinical studies on the same subject that will be conducted in the following stages. Second, more comprehensive clinical information can be achieved by analyzing the datasets obtained from multicenter medical studies to predict duodenal cancer risk with a higher probability in patients with FAP.

As a result, in the current study, genes that may be related to the development of duodenal cancer in FAP patients were identified, and genomic markers of the disease were divulged.

With more comprehensive analyses to be made in the future, the reliability of the genes obtained can be tested, treatment options relating these genes can be developed, and their applicability in medical practice can be clarified.

## Data Availability

The datasets analyzed during the current study are available from the corresponding author on reasonable request.

## Figures and Tables

**Table 1.** Comparison of Genetic Alterations in FAP Cases (with Cancer) and FAP Control (with Adenomatous Tissue) Groups Based on Tissue Analysis

Genes	Groups		P*
	Duodenal Tissue with Cancer (FAP Cases)	Duodenal Tissue with Adenoma (FAP Control)	
	Mean ± SD	Mean ± SD	
DEFA5	6.209 ± 1.121	10.343 ± 1.286	<.001
ADH1C	5.497 ± 0.805	9.373 ± 0.656	<.001
DEFA6	3.871 ± 0.687	7.65 ± 1.772	<.001
CLCA1	5.509 ± 1.143	8.948 ± 1.032	<.001
CPS1	5.097 ± 0.64	8.317 ± 0.608	<.001
APOB	3.555 ± 0.457	6.421 ± 1.209	<.001
DMBT1	8.268 ± 1.395	10.808 ± 0.617	<.001
GIP	5.495 ± 0.458	7.969 ± 1.396	<.001
ANPEP	8.164 ± 1.559	10.591 ± 0.562	<.001
SPP1	7.614 ± 1.755	4.864 ± 0.472	<.001
AC078802.1	9.616 ± 0.71	7.488 ± 1.399	<.001
LINC01962	10.581 ± 0.62	8.346 ± 1.326	<.001
VCAN-AS1	6.008 ± 1.162	3.991 ± 0.265	<.001

*Independent sample *t*-test.

**Table 3.** Comparison of Genetic Alterations in FAP Cases (with Adenoma) and FAP Control (with Adenomatous Tissue) Groups Based on Tissue Analysis

Genes	Groups		*P* ^*^
	Duodenal Tissue with Adenoma (FAP Cases)	Duodenal Tissue with Adenoma (FAP Control)	
	Mean ± SD	Mean ± SD	
APOA4	6.025 ± 1.257	7.68 ± 1.531	.008
ADH1C	7.754 ± 1.358	9.373 ± 0.656	.002
CPS1	6.75 ± 1.054	8.317 ± 0.608	<.001
CLCA1	7.398 ± 1.451	8.948 ± 1.032	.006
MIR548X	6.85 ± 1.111	8.229 ± 0.743	.002
SI	4.92 ± 0.925	6.192 ± 0.744	.001
ADH4	5.646 ± 1.026	6.894 ± 0.788	.003
LOC399753	6.41 ± 0.813	7.626 ± 0.536	<.001
DMBT1	9.596 ± 0.774	10.808 ± 0.617	<.001

^*^Independent sample *t*-test.

**Table 4.** The Result of the Performance Metrics Obtained Based on the XGboost Findings

Metric	Value (%)
Accuracy	95.8 (87.8-100)
Balanced accuracy	95.8 (87.8-100)
Sensitivity	91.7 (61.5-99.8)
Specificity	100 (73.5-100)
Positive predictive value	100 (71.5-100)
Negative predictive value	92.3 (64-99.8)
F1 score	95.7 (87.5-100)

**Table 5.** Comparison of Genetic Alterations in FAP Cases (with Normal Tissue) and FAP Control (with Adenomatous Tissue) Groups Based on Tissue Analysis

Genes	Groups		*P* ^*^
	Duodenal Tissue with Normal (FAP Cases)	Duodenal Tissue with Adenoma (FAP Control)	
	Mean ± SD	Mean ± SD	
CEACAM6	6.388 ± 0.63	8.596 ± 0.629	<.001
ANXA10	4.467 ± 0.704	6.439 ± 0.77	<.001
SLC12A2	6.35 ± 0.919	8.216 ± 0.623	<.001
OLFM4	8.513 ± 1.112	10.257 ± 0.704	<.001
SNORD78	7.094 ± 0.988	8.719 ± 0.377	<.001
LYZ	9.138 ± 1.339	10.722 ± 0.515	.002
XIST	4.729 ± 1.491	6.3 ± 2.071	.044
SNORD58A	7.802 ± 1.325	9.365 ± 0.385	.002
SNORA22	7.02 ± 1.036	8.512 ± 0.699	<.001
REG4	5.53 ± 0.527	7.009 ± 0.815	<.001
ADH1C	7.955 ± 0.957	9.373 ± 0.656	<.001
SPINK1	6.624 ± 0.505	8.002 ± 0.776	<.001
SNORD123	7.696 ± 0.631	9.063 ± 0.35	<.001
SNORD60	9.203 ± 1.198	10.544 ± 0.234	.003
CPS1	7.018 ± 0.749	8.317 ± 0.608	<.001
CEACAM5	6.204 ± 0.631	7.493 ± 0.573	<.001

^*^Independent sample *t*-test.

**Table 6.** The Result of the Performance Metrics Obtained Based on the XGboost Findings

Metric	Value (%)
Accuracy	91.7 (80.6-100)
Balanced accuracy	91.7 (80.6-100)
Sensitivity	100 (73.5-100)
Specificity	83.3 (51.6-97.9)
Positive predictive value	85.7 (57.2-98.2)
Negative predictive value	100 (69.2-100)
F1 score	92.3 (81.6-100)

**Table 2.** The Result of the Performance Metrics Obtained Based on the XGboost Findings

Metric	Value (%)
Accuracy	95.8 (87.8-100)
Balanced accuracy	95.8 (87.8-100)
Sensitivity	100 (73.5-100)
Specificity	91.7 (61.5-99.8)
Positive predictive value	92.3 (64-99.8)
Negative predictive value	100 (71.5-100)
F1 score	96 (88.2-100)
